# Rates and reasons for blood donor deferral, Shiraz, Iran. A retrospective study: Taxas e razões para o diferimento de doadores de sangue, Shiraz, Irã. Um estudo retrospectivo

**DOI:** 10.1590/1516-3180.2013.7110002

**Published:** 2014-11-28

**Authors:** Leila Kasraian, Neda Negarestani

**Affiliations:** I MD, MSc. Associate Professor and Manager of Fars Research Department Center, Blood Transfusion Research Center, High Institute for Transfusion Research and Education, Shiraz, Iran.; II MD, MSc. Assistant Professor and Manager of Shiraz Consultation Center, Iranian Blood Transfusion Research Center, Shiraz, Iran.

**Keywords:** Blood donors, Blood safety, Donor selection, Blood supply [subheading], Blood transfusion

## Abstract

**CONTEXT AND OBJECTIVE:**

Knowledge of the reasons for donor deferral can help in planning more efficient recruitment strategies and evaluating donor selection criteria. This study aimed to investigate the rates and reasons for donor deferral.

**DESIGN AND SETTING:**

Retrospective study at Shiraz Blood Transfusion Center, Shiraz, Iran.

**METHODS:**

141,820 volunteers were interviewed confidentially by physicians before blood donation. The rate of and reasons for donor deferral were investigated according to demographic characteristics. The data were analyzed using the comparison-of-proportions test of the MedCalc statistical software.

**RESULTS:**

43,839 people (30.9%) who had come for blood donation were deferred, 1,973 (4.5%) of them permanently. The deferral rate was significantly higher among women, single individuals and first-time donors, compared with men, married individuals and those with a history of previous donation (P < 0.0001). The deferral rate was significantly higher in the 17 to 30-year-old group (P < 0.05). The reasons for deferral were divided into five categories: risk factors possibly related to HIV or hepatitis (43.6%), underlying diseases (31.9%), non-eligible conditions (13.5%), medications that interfere with blood donation (7.8%) and risk factors that may relate to bacterial or viral infections except HIV and hepatitis infections (3.2%).

**CONCLUSION:**

Effective measures are required for documenting the impact of deferral on blood availability, monitoring the effectiveness of and need for deferral, and determining the reasons and rates of deferral.

## RESUMO

**CONTEXTO E OBJETIVO:**

Conhecimentos sobre os motivos do adiamento da doação de sangue podem ajudar a planejar estratégias mais eficientes de recrutamento e avaliar critérios de seleção de doadores. O objetivo foi investigar as taxas e os motivos para o adiamento da doação.

**TIPO DE ESTUDO E LOCAL:**

Estudo retrospectivo no centro de transfusão de sangue de Shiraz, Irã.

**MÉTODOS:**

141.820 voluntários foram entrevistados confidencialmente por médicos antes da doação de sangue. A taxa e as razões para o adiamento da doação foram pesquisadas de acordo com características demográficas. Os dados foram analisados com o teste de comparação de proporções do *software* estatístico MedCalc.

**RESULTADOS:**

43.839 pessoas (30.9%) encaminhadas para doação tiveram sua doação diferida, sendo 1.973 permanentemente. A taxa de adiamento foi significativamente maior entre as mulheres, solteiras e doadoras de primeira vez, em comparação com os homens, casados e os com história de doação anterior (P < 0,0001). A taxa de adiamento foi significativamente maior no grupo de 17-30 anos de idade (P < 0,05). As razões para o adiamento foram divididas em cinco categorias: fatores de risco que podem estar relacionados com HIV ou hepatite (43,6%), doenças subjacentes (31,9%), condições não elegíveis (13,5%), medicamentos que interferem na doação (7,8%) e fatores de risco que podem estar relacionados com infecções bacterianas ou virais, exceto infecções de HIV e hepatite (3,2%).

**CONCLUSÃO:**

São necessárias medidas eficazes para documentar o impacto do diferimento na disponibilidade de sangue, monitorar eficácia e necessidade de adiamento e determinar as razões e as taxas de diferimento.

## INTRODUCTION

Blood safety is a major concern all over the world. One of the most important steps used to ensure blood safety is blood donor selection.^1^^,^^2^^,^^3^^,^^4^^,^^5^ Blood donor eligibility is determined by medical interview, based on national guidelines for donor selection.^6^ Donor screening criteria are established to protect both donors and recipients.^7^ To ensure blood safety, safe donors need to be recruited and high-risk donors should be discouraged from donation.^8^

Donor selection, aimed at identifying donors who are in the window period of infectious diseases, is one of the main measures used to improve blood safety.^5^ In spite of the importance of blood donor selection for blood safety, selection processes have negative impacts on blood supply because many deferred donors do not return to donate again, because of negative feelings toward their deferral.^9^^,^^10^^,^^11^

Deferral rates and the reasons for deferral vary from one center to another.^11^ Better knowledge of the reasons and rates of donor deferral can help in designing more efficient recruitment strategies.

## OBJECTIVE

We investigated the deferral rate and the reasons for donor deferral among blood donors, according to demographic characteristics, in Shiraz (southwestern Iran).

## METHODS

This retrospective cross-sectional study was conducted from March 21, 2009, to March 21, 2010, at Shiraz Blood Transfusion Center, one of the main transfusion centers in Iran. The Institutional Ethics Review Committee of the Blood Transfusion Organization approved the study protocol. Before donation, those who had come for blood donation read brochures and pamphlets about blood safety and blood donation criteria. After reading these pamphlets, they made a decision whether to donate blood and register for donation. All individuals who had come for blood donation were interviewed by physicians, and all interviews took place in privacy. The physician assured donors that all their information would be kept confidential. The authors of this paper also made sure that the data remained confidential.

Standard operating procedures (SOP) based on national guidelines were used for donor selection and deferral. Donor hemoglobin (Hb) was checked using the HemoCue Hb 201 (HemoCue AB, Angelholm, Sweden). The cutoff point for Hb was 12.5 g/dl. The interview data were entered into the “Negare” software (version 4.56), which was developed in the Iran Blood Transfusion Organization (IBTO). At the end of the interview, acceptance or deferral of donors and the reasons for deferral were entered into the database. If the donor was deferred temporarily, the length of the deferral period in days was recorded. During the deferral period, donors were not allowed to donate. Confidential self-exclusion (CSE) is used in the center, and the physicians informed donors about this option. In accordance with the IBTO CSE policy, donors can choose that their blood should not be used, and a mailbox for their CSE forms is located at the entrance to the donation room after they are interviewed by physicians. The reasons for deferral were divided into five categories:

1) Risk factors that may relate to HIV or hepatitis infections (high-risk sexual contacts, bloodletting, intravenous drug abuse, receiving blood in year prior to donation, presence of hepatitis after the age of 11 years, presence of hepatitis in a family member, history of jaundice, tattooing, sexually transmitted diseases, previous positive results in pre-donation screening or confirmatory tests, history of jail or prison for more than 72 hours in the preceding year or history of endoscopy).
2) Underlying diseases (anemia, unsuitable blood pressure, endocrine diseases, allergy, epilepsy, diabetes *mellitus*, psychological diseases, gastrointestinal diseases, autoimmune diseases, asthma, respiratory diseases, coronary artery diseases, renal diseases, cardiopulmonary disease, metabolic diseases, surgery, rheumatological diseases, coagulation disorders, dermatological diseases, neurological diseases, malignancy, blood disorders, chemical war injuries or receipt of allograft transplant).
3) Medication used (drug, vaccination or hormone).
4) Non-eligible general condition (unsuitable criteria for donation, fasting, unsuitable blood donation or plateletpheresis or plasmapheresis interval, unsuitable weight, unsuitable age, restriction due to job, pregnancy, menstruation, breastfeeding, repeated fainting, unsuitable vein for phlebotomy or previous confidential self-exclusion).
5) Risk factors that may relate to viral or bacterial infections, except HIV and hepatitis (common cold, history of traveling to places endemic for malaria, history of malaria, fever, undergoing dental treatment, history of bacterial infections or history of viral infections).

Donors between 17 and 65 years of age were eligible for donation. All of the donors were volunteers and all of them were allogeneic blood donors. The computerized records of individuals who had come for blood donation were analyzed to estimate the types of deferral (permanent or temporary). The characteristics of deferred and accepted donors were compared. The most common reasons for deferral were determined according to the demographic characteristics (educational level, donation status, sex and age). The data were analyzed using the MedCalc statistical software, with comparison of proportions, and P values less than 0.05 were considered significant.

## RESULTS

From March 21, 2009, to March 21, 2010, 141,820 individuals came to the center for blood donation. Most of the people presenting for blood donation were male (93.74% versus 6.26%); 48.2% (68,376) were first-time donors and 22.1% (31,363) had university-level education. The characteristics of the individuals presenting for blood donation are expressed in Table 1. Out of the entire group, 43,839 (30.9%) were deferred from donation. Among the deferred donors, 4.5% (1,973) were deferred permanently.

**Table 1.**
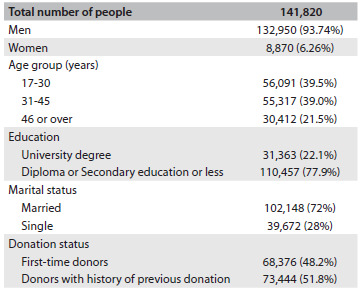
Characteristics of the individuals who came for blood donation

The deferral rates are summarized in Table 2. The deferral rate was significantly higher among women (38.6% versus 29.5%), single individuals (52.9% versus 21.07%), first-time donors (48.2% versus 13.1%) and donors with low education levels (30.7% versus 27.4%), compared with men, married individuals, those with a history of previous donation and highly educated donors (P < 0.0001). The deferral rate was also significantly higher in the 17 to 30-year-old group (P < 0.0001). The top five reasons for deferral were risk factors that might relate to HIV or hepatitis infections (43.6%), underlying diseases (31.9%), non-eligible general conditions (13.5%), medications interfering with blood donation (7.8%) and risk factors that might relate to bacterial or viral infections except for HIV and hepatitis (3.2%). Among women, 25.8% were deferred because of anemia. The top three deferral reasons according to demographic characteristics are summarized in Table 3. In this study, the most common reason for deferral among men was risk factors that might relate to HIV or hepatitis infections (43.07%) and, among women, it was underlying diseases (60.4%). In our study population, the most common leading cause of short-term deferral was risk of HIV infection (38.2%), and in our permanently deferred donors, the most frequent reasons for deferral were positive results in previous screening tests for HIV, hepatitis B or hepatitis C (22.7%). The most common reason for deferral among first-time donors was risk factors that might relate to HIV or hepatitis infections (47.74%), and among donors with a history of previous donation, it was underlying diseases (36.6%).

**Table 2.**
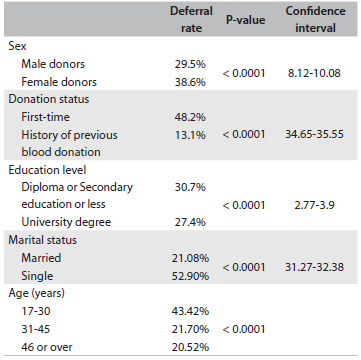
Deferral rates among donors according to demographic characteristics

**Table 3.**
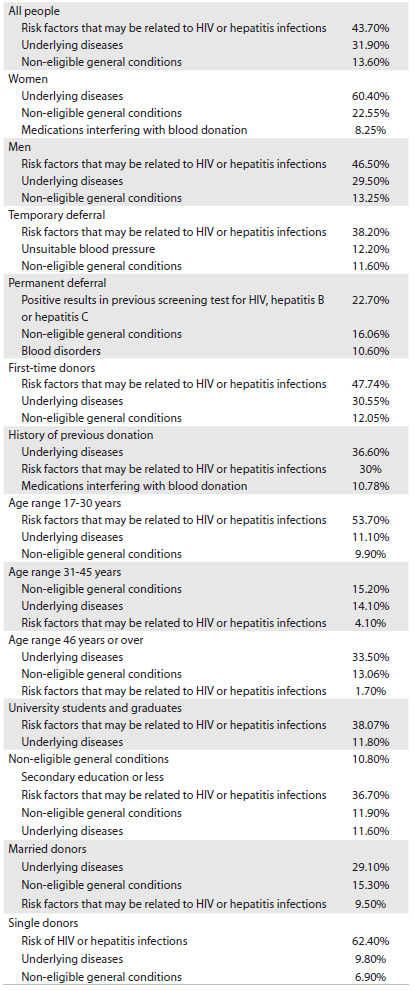
The three most frequent reasons for deferral according to demographic characteristics

## DISCUSSION

Blood donor deferral leads to loss of available blood units for transfusion. In this study, the deferral rate was 30.9%. The deferral rates elsewhere in Asia have been found to be 14.6% in Turkey;^8^ 11.6%, 16.4% and 9% in different studies in India;^10^^,^^12^^,^^13^ and 14.4% in Singapore.^14^ Reports from the United States have found deferral rates of 12.8% among a total of 47,814,370 blood donors between 2001 and 2006;^5^ 13.6% in a study on 116,165 blood donors;^15^ and 15.6% in a study on 586,159 donors.^16^ The deferral rate in a European study was 10.8%.^17^ The deferral rate in our center is much higher than other studies and even much higher than in other studies in the same geographical region, like in Turkey. All of the blood donors in our center were volunteers, but the donors in Turkey were both replacement blood donors and volunteers. The differences in deferral rate cannot be explained through this. The differences might also be because of marked differences in blood donor eligibility criteria, lack of knowledge among donors regarding the criteria for donation, greater caution among physicians in donor selection or differences in donor motivations for blood donation. Most of the donors in our centers said that they wanted to donate blood for altruistic reasons,^18^ and some of them may have come for donation in spite of medical ineligibility for blood donation. This shows the importance of educating donors about donation criteria and the deferral period. The differences in deferral rate in relation to other studies (Table 4) may have been because of changes in deferral trends over the years. Thus, further studies are needed in order to evaluate deferral rates and reasons in different parts of the world for longer periods.

**Table 4.**
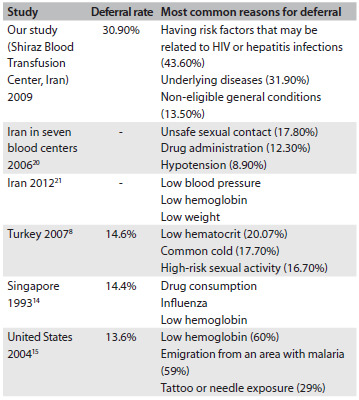
Comparison of deferral rates and the most common reasons for deferral in our study, with other studies

Some of the donor eligibility criteria in our center were different from those in other countries. For example, in our center, every sexual relationship outside of legal marriage was assumed to be an unsafe sexual contact even with use of protective devices (barrier methods like condoms). The deferral period for tattooing in our center according to our SOP is one year but, according to the American Red Cross criteria, if the tattoo was applied by a state-regulated entity using sterile needles and ink, this is acceptable for blood donation.^19^ For acupuncture, the deferral period in our center is one year but again, according to the American Red Cross criteria, this is acceptable.^19^ The deferral periods for dental procedures and dental surgery in our center are 3-8 days but, according to the American Red Cross criteria, the period is only three days.^19^ The deferral period after antibiotic injection in our center is 28 days but, according to the American Red Cross criteria, it is only 10 days.^19^ Some of the donors may come for blood donation during their deferral period and these donors may falsely increase the donor deferral rate.

Short-term deferrals accounted for 95.5% of all deferrals in our study. In a study by Custer et al., short-term deferral accounted for 68.5%;^15^ in another study by Shaz et al. it was 68%;^16^ and in a study by Lawson-Ayayi et al. in France, it was 91.3%.^17^ These findings were different from our result.

In this study, the deferral rate was higher among females (39.9% versus 30.3%). Similar figures were reported from two studies in the United States and Turkey, in which females had a higher deferral rate,^1^^,^^8^^,^^16^ while a study in France showed that greater numbers of female donors were temporarily deferred because of medical unsuitability for donation, but that there was no relationship with sex among the permanently-deferred donors.^17^ This finding was parallel to our results. The higher deferral rate among females may have been because of the higher prevalence of iron deficiency anemia in women of reproductive age.

The most frequent reasons for deferral in our study were the presence of existing risk factors which may have been related to HIV or hepatitis infections (43.6%), underlying diseases (31.9%), non-eligible general conditions (13.5%), medications interfering with blood donation (7.8%) or risk factors relating to bacterial or viral infections except HIV and hepatitis infections (3.2%). In another study in Iran between 2002 to 2004, the most frequent reasons for deferral in seven blood centers were unsafe sexual contact (17.8%), medications that interfere with blood donation (12.3%) and hypotension (8.9%).^20^ In another study by Cheraghali, which was carried out in Iran between 2002 and 2011, the most common reasons for deferral were low blood pressure, low hemoglobin and low weight.^21^ These findings were different from the reasons for deferral in our center. These differences may have been due to cultural differences, greater frequency of traveling to other countries for business reasons and presence of many universities that attract more young people for blood donation who may more frequently have high-risk behavior, stricter donor selection, more cautious donor selection or more comfort with regard to expressing high-risk behavior. In another study, the most common reasons for temporary deferral were low hemoglobin (Hb) (46%), common cold (19%) and elevated temperature (10%).^22^ In a study by Lim et al., the most frequent reasons for deferral were drug consumption, influenza, low Hb, hypertension, and recent high-risk sexual activity.^14^ In another study by Custer et al., the main reasons for deferral were low Hb (60%), emigration from an area with malaria (59%) and tattoo or needle exposure (29%).^15^ In a study on a Turkish population, the main reasons for short-term deferral were common cold in men and low hematocrit in women, while low hematocrit was the most common reason overall (20.07%), followed by common cold (17.7%), high-risk sexual activity (16.7%), hypertension (5.6%) and polycythemia (2.8%).^8^ In our study, the main reasons for deferral were risk of HIV or hepatitis infection in men (46.5%) and underlying diseases (60%), while 25.8% of the women were deferred because of anemia. This was similar to a study among Turkish donors in which the leading reasons for deferral were high-risk sexual activity (20.2%) in men and low Hb (51.6%) in women.^8^ The comparisons of deferral rates and the most common reasons for deferral in our study in relation to other studies are summarized in Table 4.

The deferral rate was higher in donors aged 17-30 years in our study, as was also found in a study in France, in which 50% of the deferred donors were less than 35 years old,^17^ whereas in a study in Turkey, the deferral rate was higher in the 50 to 65-year age group.^8^

In our study, the deferral rate was also higher among first-time donors (48.1% versus 13.1%). This may have been because repeated donors have greater awareness about blood donation criteria, and it illustrates the importance of recruiting regular donors and educating the general population regarding the eligibility criteria for blood donation.

In this study, the most common reason for deferral among first-time donors was risk factors that might be related to HIV or hepatitis infection, while among donors with a history of previous donation, it was underlying diseases. This emphasizes the importance of recruiting repeated blood donors in order to ensure blood safety.

The deferral rate because of the risk of HIV or hepatitis infections among married donors was significantly lower than among single donors (21.07% versus 52.9%) in this study. This may have been because of the greater commitment to family life and values among married donors, or denial of high-risk behavior in this group.

In spite of deferral of 30.9% of donated blood, which led to discarding of blood units, the prevalence rates of HBS antigen (Ag), HCV antibody (Ab) and HIV Ag and Ab in Shiraz blood donors during the study period were low (0.29%, 0.15% and 0.01%, respectively). In other studies, the prevalence rates of HBS Ag, HCV Ab and HIV Ab in 2006 in western Ethiopia were 25%, 13.3% and 11.7%.^23^ The prevalence rate of HBS Ag in volunteer Albanian blood donors was 7.9% in 2009.^24^ The prevalence rates of HBS Ag, HCV Ab and HIV Ab in Filipino blood donors in 2002-2004 were 4.2%, 0.3% and 0.006%.^25^ In another study in northern India, the prevalence rate of HIV Ab was 0.234%.^26^ These figures were higher than in our study, which emphasizes that strict donor selection may have led to blood safety and lower prevalence rate of HBS, HCV and HIV in Shiraz donors.

### Limitation

One of the limitations of this study was that we recorded reasons for deferral from only one source (the donor database). This source only recorded the main reason, without mentioning all the reasons for deferral, even though in some cases there may have been some different reasons. Another limitation of our study was that some deferred donors may come back during their deferral period, thereby falsely increasing the deferral rate.

Donor deferral may discourage potential donors. A large number of deferred donors do not return to donate again because of negative feelings from deferral, regarding themselves and the blood donation process.^10^^,^^11^^,^^27^ Understanding the rates and reasons for deferral can help in planning recruitment programs and in obtaining more accurate estimates of the real eligible blood donor pool, which may vary considerably from the potential blood donor pool.^10^ Riley et al. showed that because of exclusion criteria, the real population of eligible blood donors is much lower than the presumed population based on age eligibility alone (17-65 years). These authors found that by taking into consideration all the donor criteria, the real donation eligible pool may reach around 59%.^28^ It is useful to determine and merge deferral rate data from all over the country in order to estimate the real eligible donation pool at the national level and plan future recruitment efforts.

It seems that in order to ensure adequate blood supply, we must encourage donors to donate blood only for altruistic reasons.^29^ We must educate them regarding donation criteria. Physicians must give donors assurances regarding the confidentiality of donor selection so that potential donors answer the questions honestly.^30^ Physicians must also give assurances regarding the confidentially of the self-exclusion option for donors who divulge their high-risk behaviors.^30^ Blood centers must recruit first-time donors, maintain their existing donors and identify the barriers that impede donation.^29^

One of advantages of this study was the large sample size. Similar studies should be conducted in other parts of the country so that deferral rates and reasons can be identified at the national level.

## CONCLUSIONS

Overall, donor deferral in our center is higher than in other studies, which shows the importance of evaluation of the donor selection process and donation criteria so that unnecessary deferral is prevented. Donor deferral leads to loss of many people from the donation pool because of concerns regarding the safety of blood for recipients. However, the actual benefits for recipient safety remain questionable. Effective measures need to be established in order to consider the effect of deferral on donor availability and donor return, and to monitor the effectiveness and necessity of deferrals and the reasons for them. Blood centers should explain the necessary donation criteria, the reason for the deferral, length of deferral and methods for preventing deferral in the future.
